# Diagnosis and treatment of streptococcal toxic shock syndrome in the pediatric intensive care unit: a case report

**DOI:** 10.5935/0103-507X.20190068

**Published:** 2019

**Authors:** Haroldo Teófilo de Carvalho, José Roberto Fioretto, Cristiane Franco Ribeiro, Isabela Ortiz Laraia, Mario Ferreira Carpi

**Affiliations:** 1 Unidade de Terapia Intensiva Pediátrica, Hospital das Clínicas, Faculdade de Medicina de Botucatu, Universidade Estadual Paulista "Júlio de Mesquita Filho" - Botucatu (SP), Brasil.; 2 Departamento de Pediatria, Faculdade de Medicina de Botucatu, Universidade Estadual Paulista "Júlio de Mesquita Filho" - Botucatu (SP), Brasil.; 3 Unidade de Terapia Intensiva Pediátrica, Hospital Estadual de Bauru - Bauru (SP), Brasil.

**Keywords:** Shock, septic/diagnois, Shock, septic/drug therapy, Intensive care units, pediatric, Exanthema, Fasciitis, necrotizing, Penicillins/therapeutic use, Aminoglycosides/therapeutic use, Combined modality therapy

## Abstract

Among the infections caused by *Streptococcus β hemolyticus* from the Lancefield serogroup A, toxic shock syndrome is perhaps the most severe, and its mortality rate is high. Its clinical similarity to other forms of shock, especially septic shock, can often confuse the evaluator and interfere with the selection of the most appropriate therapy. This report aims to inform readers of the need to add this syndrome as a differential diagnosis in cases of shock, especially those with no well-defined clinical manifestations. For this purpose, we present the case of an infant with common flu-like symptoms who progressed rapidly with a rash, a reduced level of consciousness and clinical and laboratory signs of shock that required intensive support. In addition to cultures indicating the etiological agent, the appearance of exanthema and necrotizing fasciitis led to the diagnosis. However, less than 50% of cases present classic clinical signs of this entity. Penicillins combined with aminoglycosides are still the therapy of choice and are supported by a high level of evidence. Despite the severity of this patient's presentation, the progression was satisfactory.

## INTRODUCTION

Group A *Streptococcus pyogenes* or β-*hemolyticus* is a naturally occurring gram-positive coccus found in the airways and skin of asymptomatic individuals and is one of the most common agents in several diseases affecting the pediatric age group.^(1)^

The incidence of invasive *S. pyogenes* ranges from 0.3 to 4.8 cases per 100,000 inhabitants in developed countries. Darenberg et al. reported an 11% incidence of toxic shock syndrome (TSS) related to type A strains, of which 9.5% of cases developed necrotizing fasciitis. Its similarity to other forms of shock hinders early diagnosis and the selection of most appropriate antibiotic therapy and may delay the removal of the source of infection.^(1,2)^

TSS is a systemic inflammatory process induced by immune mediators in response to certain infections caused by these agents, in which exotoxins act as superantigens, inducers of proliferation and activation of T lymphocytes and macrophages because they stimulate, without specificity, nearly 20% of the total population of T cells to bind directly to their receptors, instead of presenting to the major histocompatibility complex (MHC); this results in the massive release of cytokines, causing tissue injury and increased capillary permeability culminating in the dysfunction of multiple organs and shock, in addition to increasing the susceptibility to endotoxic shock due to gram*-*negative bacteria.^(3,4)^

This specificity is conferred by a streptococcal surface protein (M protein) that confers a high degree of resistance to heat and acidic pH and is responsible for promoting fixation in human epithelial cells, preventing complement opsonization and phagocytosis. Subtypes 1, 3 and 18 are related to invasive infections with a worse prognosis, reaching 50% mortality in cases that progress to TSS.^(5,6)^

Concentrated outbreaks have been reported since the first description of the disease 90 years ago and are related to reemerging conditions and soft tissue and respiratory tract infections, usually preceded by common and less-specific flu-like symptoms.

This report aimed to describe the peculiar characteristics of the syndrome, considering its severity, and to address need for specific and differential diagnoses to determine therapeutic targeting, remove the source of infection, and avoid the indiscriminate use of antibiotics in intensive care units. The legal guardian of the patient authorized the use of data from the medical records and the photos taken during hospitalization to prepare this report by signing the Informed Consent Form, which was subsequently submitted to the Research Ethics Committee through the Brazil Platform.

## CASE PRESENTATION

**Figure 1:**
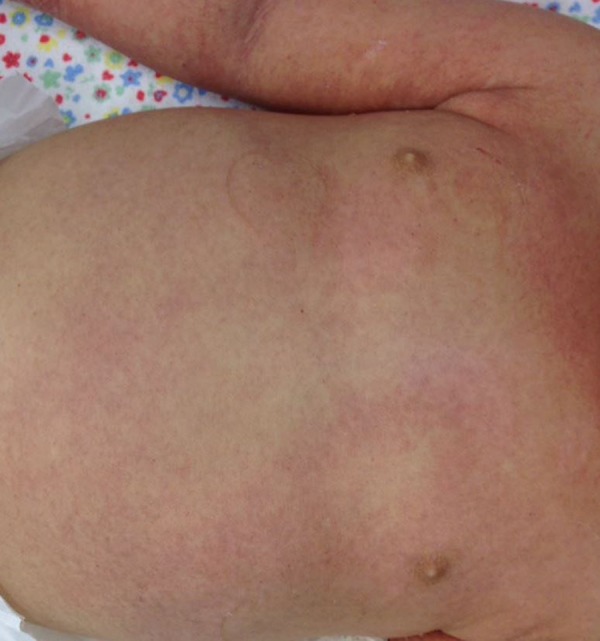
Thorax and abdomen with diffuse erythematous rash.

**Figure 2:**
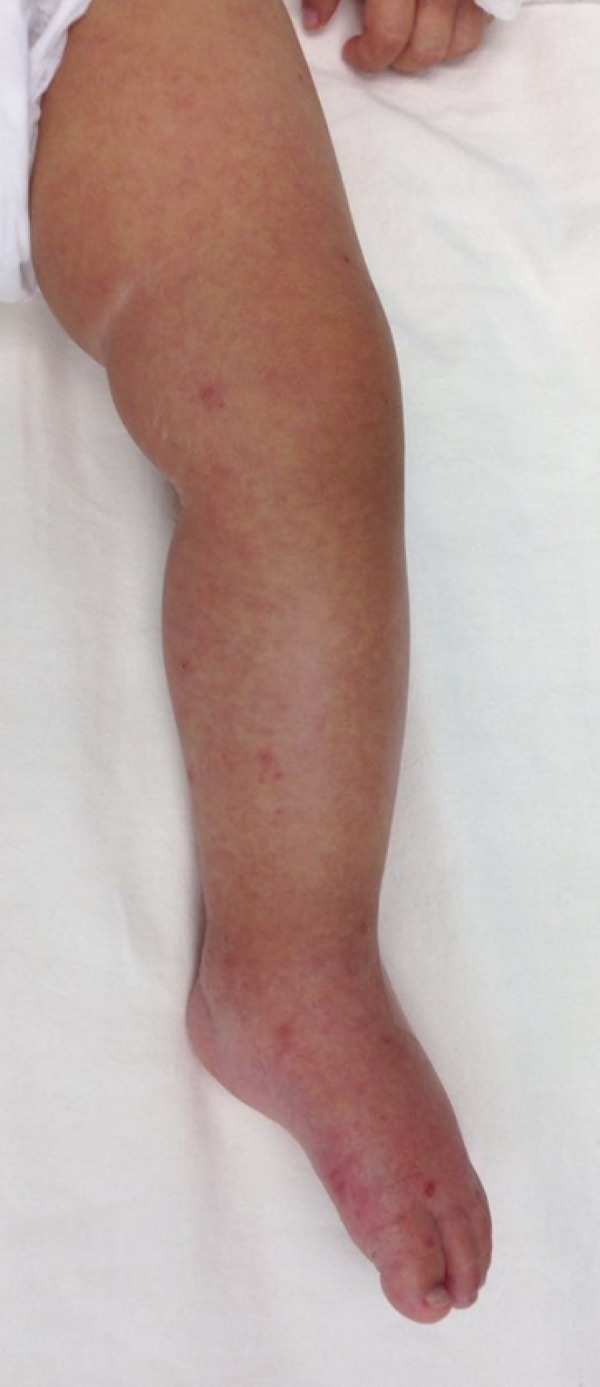
Lower left limb with diffuse erythematous rash.

A young female infant aged 1 year and 2 months, 9kg, previously healthy and eutrophic, presented hyaline coryza, nasal obstruction, dry cough and continuous fever between 38.5 and 39.4ºC and had been receiving antipyretics for 5 days. The rash emerged on the second day of symptoms and was accompanied by a decline in the general condition. The patient was admitted to the pediatric ward for investigation (Figures 1 and 2).

At the admission examination, the patient was in a regular general condition and was jaundiced, dehydrated and febrile, with generalized edema of the upper and lower limbs and face. Diffuse maculopapular exanthema was present on the lower and upper limbs, trunk, face, hands and feet; was rough, pruritic, with areas of petechiae; and more evident on the left arm and right leg.

Otoscopy found that the left auditory canal was hyperemic and the tympanic membrane was opaque, with a moderate quantity of fetid serous effusion. Oroscopy showed grade II tonsils, with mild hyperemia of the pillars without exudate. Cardiac auscultation detected a galloping heartbeat with a holosystolic murmur (3+/6+) in the mitral focus and tachycardia; blood pressure was 108 × 56mmHg. Lungs presented a bilaterally distributed vesicular murmur, coarse crepitations at the left base, no discomfort with eupnea and 95% saturation in room air. The liver was palpated 4cm from the right costal margin. Glasgow score was 11 with little contact and no neck stiffness or focal neurologic deficits.

On the ward, empiric antimicrobial treatment with amoxicillin and clavulanate at a dose of 100mg/kg/day was established according to the routine of the service. At the end of the first day of hospitalization, the patient evolved, developing toxemia, tachycardia, tachypnea, hypotension and prolonged capillary refill time, and the hypothesis of septic shock with an undefined source was suggested. The patient received a 20mL/kg crystalloid expansion in a single aliquot and was referred to the pediatric intensive care unit (ICU).

Upon admission to the ICU, functional echocardiography was performed, and the results indicated reduced myocardial contractility with consequent decrease of the ejection fraction, followed by ultrasound-guided hemodynamic state assessment. Two new expansions were indicated (totaling 60mL/kg) and continuous epinephrine was initiated and was titrated up to 0.3mcg/kg/minute. The patient was intubated, and a central venous access was placed to allow access for invasive blood pressure monitoring.

After a retrospective case analysis of the rapid progression, the presence of a diffusely distributed rash, flu prodromes and shock, we considered the possibility of TSS, replacing the amoxicillin and clavulanate with ceftriaxone 100mg/kg/day and clindamycin 20mg/kg/day. A CT scan of the patient's ears showed soft-tissue visibility in the middle ear bilaterally with a normal mastoid without lytic lesions, suggesting acute otitis media.

After the initial shock support measures were provided, the participant maintained hypotension and central venous saturation < 70%; norepinephrine 1mg/kg/ min followed by hydrocortisone 100m/m2 body surface were administered. On the second day of ICU admission, the patient developed multiple organ dysfunction requiring blood transfusion (red blood cells and platelets) and vitamin K. The bedside transthoracic echocardiogram showed no intracardiac vegetation.

**Figure 3:**
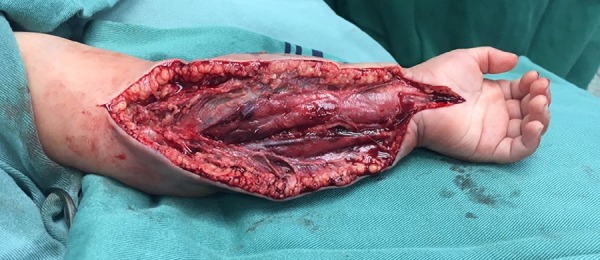
Bedside fasciotomy due to necrotizing fasciitis.

**Figure 4:**
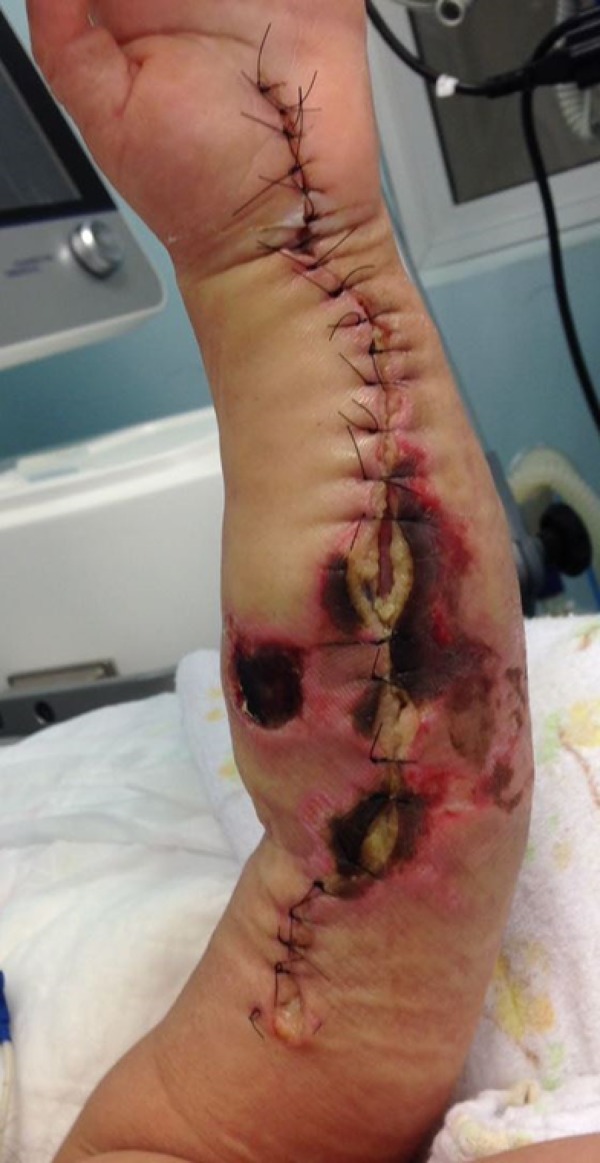
Upper limb left after suture of the fasciotomy due to necrotizing fasciitis.

The patient progressed with hyperemia and generalized edema of the left upper limb, with a capillary refill time > 5 seconds and a weak radial artery pulse, diffuse petechiae and bruising in the lateral region of the contralateral limb suggesting necrotizing fasciitis, and bedside fasciotomy was performed. Cultures of the skin, subcutaneous tissue and muscles were collected (Figures 3 and 4).

Due to the severity of the condition and the worsening of the hematological and white blood cell status on the third day of hospitalization, the medical team had a clinical meeting and chose to schedule antibiotic therapy, suspending ceftriaxone and maintaining clindamycin combined with cefepime and vancomycin.

The results of the cultures collected at admission indicated only the presence of *S. pyogenes* in the otological secretions of the left ear. This ear was suppurative on the fourth day, when it was possible to titrate the vasoactive drug and the steroid, maintaining only 0.2mcg/kg/minute of epinephrine suspended together with vancomycin on the fifth day of intensive care.

The invasive ventilatory support was removed, followed by discharge to complete the therapeutic regimen on the ward from the sixth day of hospitalization. The patient remained afebrile, with furfuraceous desquamation of the limbs associated with intense pruritus in the folds. She was discharged from the hospital on the 14^th^ day after completing treatment the cefepime and clindamycin regimen.

## DISCUSSION

Scarlet fever and sepsis during the puerperal period are historically the main causes of morbidity and mortality related to streptococcal TSS; they reached mortality rates of 30% at the end of the 19th century. After the 1900s, the incidence rate did not reach 2%; however, in the 1980s, there was a resurgence of severe infections caused by *S. pyogenes,* with a return to the high incidence and mortality reported in the previous century. This variation can be explained by the natural variation in pathogen virulence.^(7)^

As highlighted in table 1, the signs and symptoms comprising the diagnostic criteria for a syndrome attributable to *S. pyogenes* are complex given that the need for a positive culture to determine the agent may hinder diagnosis and may underreport infection, either because of the sensitivity and specificity of the culture method used, the inappropriate harvesting or storage of cultures and the introduction of antimicrobial therapy prior to sample collection.

**Table 1:** Streptococcal toxic shock syndrome: case definition (CDC 2011)

Fever	Greater than or equal to 38.9 ºC
Rash	Diffuse macular erythema
Desquamation	1 - 2 weeks after the start of the rash
Hypotension	Below the 5^th^ percentile for age
Multisystemic involvement	Gastrointestinal: vomiting or diarrhea at the onset of disease
	Muscular: severe myalgia or creatine kinase MB isoenzyme (CMPK) level at least twice the upper limit of normal
	Mucous membrane: vaginal, oropharyngeal or conjunctival hyperemia
	Renal: urea and creatinine values at least twice the upper limit of normal to age
	Hepatic: total bilirubin, GOT and GPT values at least twice the upper limit of normal for age
	Hematologic: platelets ≤ 100,000/mm^3^
	Neurological: disorientation or changes in the level of consciousness without focal neurological signs (when fever and hypotension are absent)
Isolation of *Streptococcus pyogenes*	Sterile site (blood, cerebrospinal fluid, peritoneal fluid and biopsy tissue)
	Nonsterile site (oropharynx, sputum, vagina and surgical site)
Case classification	
Probable: case that meets the clinical criteria in the absence of another etiology that might explain the clinical picture and the isolation of group A *Streptococcus* from a nonsterile site	
Confirmed: case that meets the clinical criteria and with the isolation of group A *Streptococcus* from a normally sterile site	

Source: adapted from the Centers for Disease Control and Prevention. Toxic Shock Syndrome (Other Than Streptococcal): 2011 Case definition [internet]. [cited 2018 Mar 12]. Available from: https://wwwn.cdc.gov/nndss/conditions/toxic-shock-syndrome-other-than-streptococcal/case-definition/2011/.^(8)^

CPMK - creatine kinase MB isoenzyme; GOT - glutamic-oxaloacetic transaminase; GPT - pyruvic glutamic transaminase.

As in the case described, approximately 20% of patients experience a flu-like syndrome characterized by fever, myalgia, nausea, vomiting and diarrhea. Localized or diffuse abrupt and severe pain that precedes the inflammatory symptoms, together with the observation of an entryway for infection, are the main characteristics described in the literature.^(9)^

Thus, organ dysfunction and shock, although similar to the presentation of septic shock, have specific clinical manifestations, such as rash, desquamation, gastrointestinal symptoms, muscle involvement, pharyngeal and conjunctival hyperemia, and renal failure, with rapid and aggressive progression.

Necrotizing fasciitis has often been associated with TSS. It is a diagnosis that should always be considered when a child exhibits erythema, heat and induration of the skin and soft tissues in association with persistent fever. Initial skin manifestations may hinder the diagnosis of necrotizing fasciitis; however, rapid progression and extension of the lesion, paresthesia, bruising, crackles, blistering or necrosis suggest the diagnosis.^(10)^

Due to reports of a clinical syndrome similar to the toxic shock induced by *Staphylococcus aureus* in 1993, an international working group linked to the Centers for Disease Control and Prevention (CDC) elaborated clinical and laboratory criteria to standardize the etiological diagnosis and provide support for specific treatment (Table 2).^(8)^

**Table 2:** Difference between staphylococcal and streptococcal toxic shock syndrome

Features	Staphylococcal TSS	Streptococcal TSS
Exotoxins	TSST-1, SEs A, B, C, D, E	SPEs A, G, H, J, SSA, MF, SMEZ
Predisposing factors	Tampon, burns and wounds	Varicella, NSAIDs and wounds
Associated infection sites	Impetigo, burns, rashes, surgical wound	Abscesses, myositis, fasciitis, surgical wound
Infection of soft tissues	Rare	Common
Acute pain	Rare	Common
Rash	Very common	Less common
Diarrhea and vomiting	Very common	Less common
Elevation of CPK	Rare	Common
Bacteremia	< 5%	60%
Desquamation	7 to 14 days	Less common
Mortality	3 to 5%	5 to 10%

Source: adapted from the Centers for Disease Control and Prevention. Toxic Shock Syndrome (Other Than Streptococcal): 2011 Case definition [internet]. [cited 2018 Mar 12]. Available from: https://wwwn.cdc.gov/nndss/conditions/toxic-shock-syndrome-other-than-streptococcal/case-definition/2011/.^(8)^

TSS - toxic shock syndrome; TSST-1 - toxin from toxic shock syndrome; SEs - staphylococal superantigens; SPE - streptococcal superantigens; NSAIDs - nonsteroidal anti-inflammatory drugs; CPK - creatine phosphokinase.

The rapid institution of antibiotic therapy and fluid resuscitation are necessary and, when possible, should be guided by the epidemiological pattern of the unit and by noninvasive hemodynamic monitoring. Vasopressor, inotropic and respiratory support should be considered, as in any other form of shock.

Knowledge of the pathophysiology of this entity and the establishment of the most appropriate antibiotic for the condition are of great importance. Penicillin is still described as the treatment of choice, but in animal models with streptococcal myositis, there was a decrease in its efficacy given the low rate of replication of group A *S.* β *hemolyticus* when the inoculum is large. Therefore, the current recommended initial therapy is the combination of a beta-lactam antibiotic and some aminoglycosides.^(11)^

Clindamycin is a semisynthetic antibiotic produced by the replacement of the 7 (R)-hydroxy group of a derivative of lincomycin, which binds to the 50S subunit of bacterial ribosomes and p the formation of peptide bonds, hindering the growth, reproduction and release of TSS-inducing toxins.^(12)^

The use of antibiotics during the acute phase of the disease eradicates the pathogen and prevents recurrence. Passive immunization with intravenous immunoglobulin is useful in severe cases and acts for weeks but does not induce active immunity.^(11,12)^

## CONCLUSION

We emphasize the importance of knowing the diagnostic criteria for this syndrome and its various spectra of presentation to establish appropriate antibiotic therapy. The initial shock support follows the same indications recommended for the other types of shock, but attention should be paid to the rapid evolution of the condition and the absence of clear foci of infection. Complications, such as the necrotizing fasciitis presented in this case, should be quickly resolved because they may contribute to hemodynamic imbalance in the patient.
